# Evaluation of Process Parameters in the Development of Ternary Ketoprofen Amorphous Solid Dispersions via Hot Melt Extrusion

**DOI:** 10.3390/pharmaceutics18020241

**Published:** 2026-02-14

**Authors:** Ana Stjepanović, Nemanja Todorović, Mihalj Poša, Ivana Marinković, Ivan Ristić, Zita Farkaš Agatić, Mladena Lalić-Popović

**Affiliations:** 1 Department of Pharmacy, Faculty of Medicine Novi Sad, University of Novi Sad, 21000 Novi Sad, Serbia; stjepanovicana94@gmail.com (A.S.); mihalj.posa@mf.uns.ac.rs (M.P.); ivanamarinkovic200@gmail.com (I.M.); zita.farkas@mf.uns.ac.rs (Z.F.A.); mladena.lalic-popovic@mf.uns.ac.rs (M.L.-P.); 2 Faculty of Technology Novi Sad, University of Novi Sad, 21000 Novi Sad, Serbia; ivan.ristic@uns.ac.rs; 3 Centre for Medical and Pharmaceutical Investigations and Quality Control (CEMPhIC), Faculty of Medicine Novi Sad, University of Novi Sad, 21000 Novi Sad, Serbia

**Keywords:** ketoprofen, solubility enhancement, dissolution, poloxamer, amorphous solid dispersion

## Abstract

**Background/Objectives**: Poor aqueous solubility of active pharmaceutical ingredients (APIs) remains a critical barrier to effective oral formulation. This study investigated the production of ketoprofen amorphous solid dispersions (ASDs) via hot melt extrusion (HME) using hydrophilic carriers and surfactants to enhance solubility and dissolution. **Methods**: ASDs were prepared by the fusion method employing mannitol or polyethylene glycol (PEG) 4000 hydrophilic carriers and further modified by addition of poloxamer 188 or poloxamer 407 as surfactants. Solubility was evaluated, and the best performing formulations were selected for HME to assess the effect of extrusion parameters (temperature, screw speed and re-extrusion) on API solubility and dissolution. Selected ASD extrudates were formulated into tablets and capsules and further tested. **Results**: Ternary ASDs exhibited higher solubility than their binary counterparts. The combinations of high-concentration hydrophilic carrier (mannitol or PEG 4000) and poloxamer 407 proved the most effective. The HME-produced ASDs showed superior solubility compared to the simple fusion method, with temperature being the most critical processing parameter, while screw speed and re-extrusion were carrier dependent, enhancing solubility for mannitol-based ASDs but not for PEG 4000; re-extrusion also led to mild color changes and technological issues preventing further processing. The selected ASD extrudates were successfully formulated into tablets and capsules with good physical characteristics and dissolution profiles. **Conclusions**: These findings demonstrate the need to further investigate the potential of re-extrusion strategies and surfactant-enhanced ASD systems for improving the oral delivery of poorly soluble drugs.

## 1. Introduction

The solubility of an active pharmaceutical ingredient (API) significantly influences the absorption process, bioavailability and the desired therapeutic effect of the medicinal product. Poor solubility presents a challenge for approximately 40% of marketed APIs and up to 90% of new drug candidates [1,2], while low bioavailability resulting from poor solubility is one of the leading causes of failure in the early stages of clinical trials [1,3]. For these reasons, considerable efforts are being directed toward the development of various techniques aimed at enhancing the solubility of poorly soluble APIs, among which the use of amorphous solid dispersions (ASDs) has emerged as a promising strategy due to its efficiency and applicability in the pharmaceutical industry [1,4].

ASDs involve mixing or dispersion in the solid state of poorly soluble, hydrophobic API within a hydrophilic inert carrier or matrix [1,4,5]. Various crystalline carriers (such as urea, sucrose, dextrose, mannitol, etc.) and polymers (including polyvinylpyrrolidone, polyethylene glycol (PEG), hydroxypropyl methylcellulose, etc.) have been employed and investigated for the preparation of ASDs [1,2,4]. Additionally, surfactants are often incorporated into these systems to produce ternary ASDs with improved characteristics and performance [1,4,6,7].

The exact mechanism of action of ASDs is not fully understood. However, it is generally believed that the increased solubility results from the transformation of the API from a crystalline to an amorphous state and its dispersion within the carrier which leads to a reduction in particle size and an increase in the surface area in contact with the dissolution medium [1,4]. The stability of ASDs and the efficiency of solubility and dissolution enhancement largely depend on the structure and physicochemical properties of the system components, as well as on the type and intensity of the interactions established between them [1,8]. Reaggregation and recrystallization of the active substance from the hydrophilic carrier upon contact with the dissolution medium or during storage have been observed. This poses a challenge, as it can significantly slow down the dissolution process [1,4]. To mitigate these issues, ternary amorphous solid dispersions (ASDs) have been investigated, as the addition of a surfactant can enhance the wetting and solubilization of the API, reducing the likelihood of reaggregation or recrystallization [1,4,6,7]. These ternary systems have been shown to significantly improve stability and/or dissolution rate of some ASDs of poorly soluble APIs such as ketoprofen, domperidone and lansoprazole [9,10,11].

A variety of methods exist for the preparation of ASDs, but they are most commonly produced using either the fusion method or the solvent-based method [1,4]. Hot melt extrusion (HME) is a contemporary fusion-based technique in which a powder blend is conveyed through an extruder by rotating screws under elevated temperature and pressure resulting in melting and intense mixing of the components [4,12]. HME offers numerous advantages, as it represents a continuous, efficient, simple and cost-effective approach for producing ASDs, with applicability in the pharmaceutical industry [1,4,12]. However, careful consideration must be given to the potential impact of various processing parameters on the properties of the resulting ASD extrudates [12]. In addition to these considerations, the application of ASDs in the development of final dosage forms is further limited by potential technological issues such as poor powder flowability and compressibility, phase separation during processing or phase separation and slow disintegration due to polymer gelling [13,14].

Ketoprofen belongs to class II of the Biopharmaceutics Classification System (BCS). It is characterized by high permeability (readily crossing biological membranes) and low, pH-dependent solubility, which negatively affects the dissolution rate from pharmaceutical dosage forms and leads to prolonged absorption time and delayed onset of therapeutic effect [5,6,15]. For APIs such as ketoprofen, improving solubility is one of the key strategies for enhancing dissolution and accelerating absorption and onset of action. Owing to its poor aqueous solubility, well-defined physicochemical profile and relatively low therapeutic dose, ketoprofen represents a suitable model API for investigating ASD systems.

Since the stability of ASDs and their effect on solubility enhancement largely depend on the structure and physicochemical properties of the API, the hydrophilic carrier and the surfactant, as well as on their proportions and the conditions under which the ASDs are prepared, investigating the influence of these factors is essential for formulating ternary ASDs with optimal characteristics [1,4,5,6,7]. In this work, we aimed to formulate and prepare binary and ternary ASDs, initially using the fusion method to optimize composition and component ratios and subsequently applying the HME method. This study evaluated the influence of formulation composition and HME processing parameters on the solubility enhancement and dissolution rate of the API. Most suitable ASD extrudates were formulated into powder blends for tablet and capsule preparation, which were evaluated in terms of physical characteristics and dissolution rate to further assess ASD processability and performance.

## 2. Materials and Methods

### 2.1. Materials

The following chemicals were used in the experiment: ketoprofen (Farmalabor, Canosa di Puglia, Italy, meets USP requirements), mannitol (Farmalabor, Canosa di Puglia, Italy), PEG 4000 (Alfa Aesar, Ward Hill, MA, USA), poloxamer 188 (BASF Chemtrade GmbH, Burgbernheim, Germany), poloxamer 407 (BASF Chemtrade GmbH, Burgbernheim, Germany). Size 0 hard gelatin capsules (Farmalabor, Canosa di Puglia, Italy; composition: indigo FD&C Blue 2 (E132), titanium dioxide, yellow iron oxide (E172), gelatin) were used for the purpose of ASD extrudates dissolution rate testing and preparation of final capsule dosage form. Tablets were prepared using anhydrous lactose (Super Tab 21AN, DFE Pharma, Goch, Germany, donated by Galenika AD, Belgrade, Serbia), magnesium stearate (Mosselman, Ghlin, Belgium), sodium starch glycolate (Primojel^®^, DFE Pharma, Goch, Germany; donated by Galenika AD, Belgrade, Serbia). Lactose (Capsulac^®^, Meggle, Wasserburg am Inn, Germany) was used as a filler in the capsule formulations. Methanol (Lachner, Neratovice, Czech Republic) was used for ketoprofen content determination. Purified water for the experiments was obtained by distillation (AC-L8, Optic Ivymen System, J.P. Selecta, Cham, Switzerland) at the Department of Pharmacy of the Faculty of Medicine Novi Sad.

### 2.2. Methods

#### 2.2.1. Preparation of ASDs by the Fusion Method

For the preparation of ASDs, ketoprofen was selected as a model BCS class II drug [15]. PEG 4000 was chosen as a second-generation amorphous polymer carrier, while mannitol served as a first-generation crystalline hydrophilic carrier for comparison [4,16,17]. Poloxamer 188 or 407 was included as a third component due to their reported ability to enhance solubility and dissolution when incorporated as surfactants in ASDs [18,19,20]. Considering that the ASDs would be prepared at elevated temperatures, the selection of these components also took into account their thermal stability reported in the literature [21,22,23,24,25]. ASDs were prepared from each tested physical mixture (PM). The individual components were accurately weighed on a technical scale (Radwag, Radom, Poland) and mixed in a powder mixer (Farmalabor, Canosa di Puglia, Italy) for 5 min at a speed of 130 rpm. Mixing was performed in a container filled to approximately 50% of its volume, using 20 g batches. The PMs were transferred to a metal dish and melted on a heating device (Boeco, Hamburg, Germany) until the mixture turned transparent, then cooled to −20 °C. The prepared binary and ternary ASDs were further investigated along with the corresponding PMs to enable direct comparison of the effects of carrier–surfactant combinations on the resulting properties. The composition of the produced binary and ternary PMs and ASDs is shown in Table 1.

#### 2.2.2. Solubility Study

PMs and ASDs corresponding to 100 mg of ketoprofen were weighed on an analytical scale (Radwag, Radom, Poland) and transferred to test tubes with 10 mL of distilled water. Solubility was tested at 25 °C and 37 °C in a thermostatic water bath with a shaker (WSB-18, Witeg Labortechnik, Wertheim, Germany). After 24 h of stirring at 150 rpm, the samples were filtered through 0.45 µm cellulose acetate membrane filters (Sartorius Lab Instruments, Goettingen, Germany) and diluted, and the concentration of ketoprofen was determined spectrophotometrically (G1103A, Agilent Technologies, Santa Clara, CA, USA) at 260 nm (Section 2.2.18) [26,27].

#### 2.2.3. Calculation of Gibbs Free Energy (∆G°_tr_)

Gibbs free energy (∆G°_tr_) represents the function of energy formation during the transformation of ketoprofen from insoluble to soluble form. ΔG°_tr_ represents the difference between the Gibbs free energy of intermolecular interactions of the hydrophobic drug in aqueous additive solution (ΔG°_in(w)_), where the additives can be polymeric surfactants, amphiphilic polymers, and similar solubilizing agents, and the Gibbs free energy of the hydrophobic drug in the ASD (ΔG°_in(SD)_), which corresponds to the thermodynamic destabilization of the drug relative to its crystalline reference state (Equation (1)) [28]:

∆G°_tr_ = ΔG°_in(w)_ − ΔG°_in(SD)_ (1)

The Gibbs free energy ΔG°_in(w)_ is always negative, while the thermodynamic destabilization of the hydrophobic drug in the amorphous state relative to the crystalline state, ΔG°_in(SD)_, is always positive.

More negative values of ∆G°_tr_ indicate better solubilization characteristics. The calculation of ∆G°_tr_ was done using the formula given in Equation (2):

∆G°_tr_ = −2.303 × R × T × log(S_S_/S_0_) (2)

where R—gas constant (8.31 J/(mol × K)), T—temperature in K, S_S_—solubility of ketoprofen from the tested systems in water and S_0_—solubility of pure ketoprofen in water.

The standard molar Gibbs free energy (ΔG°_tr_) represents a thermodynamic measure of the increased spontaneity associated with the solubilization of hydrophobic drug particles from an ASD, relative to the solubilization of crystalline hydrophobic drug particles taken as the reference state.

#### 2.2.4. Determination of Melting Point

The melting points of the pure substances and selected PMs were determined to adjust the temperature for the HME. A micromethod was applied using small amounts of samples with the use of capillary tubes. Before testing, the samples were kept in a desiccator with silica gel for 24 h. The melting point was determined on a melting point apparatus (Electrothermal AZ9003, Staffordshire, UK) at a heating rate of 1 °C/min, and melting was observed visually. The test was performed in triplicate, and the results were expressed as the interval of the lowest and highest temperature of three measurements.

#### 2.2.5. Preparation of ASDs by the HME Method

The solubility study results of the prepared formulations (PMs and Fs) were used to determine the optimal ASD composition for each carrier which were prepared for HME. PMs corresponding to the formulations containing mannitol (E1–E8) and PEG 4000 (E9–E16) were dried in an oven (Memmert, Schwabach, Germany) at 40 °C for 24 h prior to extrusion [29]. HME was performed using a single-screw extruder (Noztek Touch, Shoreham, West Sussex, UK) under varying process parameters, including extrusion temperature, as summarized in Table 2.

#### 2.2.6. Solubility Study and Calculation of Gibbs Free Energy (∆G°_tr_) for ASDs Prepared by HME

Solubility study at 37 °C and calculation of Gibbs free energy were conducted for ASDs prepared by HME using the previously described methods.

#### 2.2.7. Dissolution Rate Testing of ASDs Obtained by HME

The dissolution rate of ASDs prepared via HME (formulations E1–E16) was evaluated. Owing to their brittle nature, the ASD extrudates were manually pressed through a 355 µm sieve to obtain uniform mass distribution. Quantities equivalent to 25 mg of ketoprofen were accurately weighed using an analytical scale (Radwag, Radom, Poland) and transferred to size 0 hard gelatin capsules. The test was performed on a dissolution apparatus with baskets (DT 800 LH, Erweka^®^, Heussenstam, Germany) at a rotation speed of 100 rpm. The test was performed in triplicate at 37 °C in 900 mL of distilled water, without maintaining sink conditions. Samples (5 mL) were withdrawn at 5, 15, 25, 35, 45 and 60 min from the start of the test and filtered through cellulose acetate membrane filter, 0.45 µm (LLG, Meckenheim, Germany). The concentration of ketoprofen was determined spectrophotometrically (G1103A, Agilent Technologies, Santa Clara, CA, USA) at 260 nm (Section 2.2.18) [26,27].

#### 2.2.8. Fourier Transform Infrared (FTIR) Spectroscopy

Fourier Transform Infrared (FTIR) analysis was carried out on the selected PMs and ASDs, as well as the corresponding pure components using a Nicolet IS10 FTIR spectrophotometer (Thermo Scientific, Waltham, MA, USA). Spectral data were acquired and processed with Omnic 8.1 software (Thermo Scientific, Waltham, MA, USA). Each sample was scanned 32 times over a range of 4000–400 cm^−1^ with a spectral resolution of 4 cm^−1^. A background spectrum was recorded prior to each measurement. The resulting spectra were subsequently plotted for comparison.

#### 2.2.9. Differential Scanning Calorimetry (DSC)

Differential Scanning Calorimetry (DSC) was performed to evaluate the thermal properties of selected PMs, ASDs and pure components using a DSC Q20 instrument (TA Instruments, New Castle, USA). Samples weighing 3–5 mg were hermetically sealed in aluminum pans and heated from 30 °C to 200 °C at a rate of 10 °C/min. Indium was employed for instrument calibration, and the sensitivity was set to 10 µV/cm.

#### 2.2.10. Tablet Blend Formulation

The best-performing ASD extrudates, in terms of solubility and dissolution enhancement as well as processability (visual appearance, consistency and stickiness), prepared by HME for each hydrophilic carrier were briefly crushed using a mortar and pestle, sieved through a 355 µm sieve and subsequently blended with excipients for tablet compression. The powder blends for tableting (T1 with mannitol and T2 with PEG 4000 as hydrophilic carrier) were prepared using a powder mixer (Farmalabor, Canosa di Puglia, Italy). All components, except magnesium stearate, were mixed for 25 min at a speed of 130 rpm (mixing intensity 5/5) in a plastic container filled to approximately 50% of its volume. After the initial mixing, magnesium stearate was added to the blend, and mixing was continued under the same conditions for an additional 2 min. The compositions of the tablet blend formulations are presented in Table 3.

#### 2.2.11. Capsule Blend Formulation

The ASD extrudates selected for tablet compression were also evaluated in capsule formulation. Thus, two capsule formulations were prepared, one for each hydrophilic carrier: K1 containing mannitol and K2 containing PEG 4000, as presented in Table 3. The amount of ASD extrudates equivalent to 25 mg of ketoprofen per capsule was calculated. Powder blend sufficient for filling 30 capsules was prepared by transferring the required quantity of ASD into a measuring cylinder and adding lactose to reach the total volume necessary for 30 capsules (i.e., 20.4 mL). The resulting powder blend was mixed in a laboratory tumbling mixer for 10 min.

#### 2.2.12. Determination of the Flowability Parameters

The bulk and tapped volumes of ASD extrudates selected for final formulations and prepared blends intended for tableting/capsulation were measured using a jolting volumeter STAV II (J. Engelsmann AG, Ludwigshafen, Germany). A 50 mL graduated cylinder was used, and the sample mass was 30 g. Bulk volume (V_0_) and tapped volume after 10, 100, 500, 900 and 1250 taps (V_f_) were recorded. All measurements were performed in triplicate, and the results were expressed as mean values ± standard deviation. The Hausner ratio (Equation (3)) and compressibility index (Equation (4)), as well as the flowability classification, were calculated according to the guidelines of the 11th edition of the European Pharmacopoeia (Ph. Eur. 11) [30]:

Hausner ratio = V_0_/V_f_ (3)

Compressibility Index = 100 × (V_0_ − V_f_)/V_0_ (4)

The angles of repose of the same formulations were evaluated. A glass funnel was placed on a laboratory tripod so that its outlet was positioned approximately 2 cm above the working surface and the paper below. The tested powders were allowed to freely fall through the funnel in amount sufficient to form a cone with a height of approximately 2 cm (real height was recorded for each measurement). The diameter of the cone base was measured, and the angle of repose was calculated using Equation (5):

tan(α) = h/(0.5 × D)(5)

where α—angle of repose, h—height of the cone and D—measured diameter of the cone base.

The test was performed in triplicate, and the results were reported as mean values ± standard deviation. Flowability was assessed according to the criteria of Ph. Eur. 11 [30].

#### 2.2.13. Preparation of Tablets and Capsules

The formulated tablet and capsule blends (Table 3) were then used for preparation of tablets and capsules.

Tablets were prepared using an eccentric tablet press (Korsch, Berlin, Germany) with a 12 mm die. Each tablet contained 25 mg of ketoprofen, with the lower punch adjusted to accommodate 0.50 g of blend, ensuring the desired drug dose. The upper punch pressure was optimized to obtain tablets of acceptable quality at the lowest possible compression force [31,32]. The tablet thickness, diameter and hardness were subsequently measured, and the results were reported as mean ± standard deviation. The prepared capsule powder blend was filled into size 0 gelatin capsules using a semi-automatic capsule filling machine (Farmalabor, Canosa di Puglia, Italy). The tablets and capsules were stored in closed plastic polypropylene containers, under room temperature and protected from light, until further testing.

#### 2.2.14. Uniformity of Mass

The uniformity of mass test was performed in accordance with the requirements of the Uniformity of mass of single-dose preparations (2.9.5.) from Ph. Eur. 11 [30] using an analytical scale (Radwag, Radom, Poland). Twenty randomly selected tablets were dusted and individually weighed on the analytical scale, and the average mass was calculated. Twenty randomly selected capsules were weighed intact. After emptying, the shells were weighed. The mass of the capsule content was obtained by difference between the two weighings, and the average content mass was calculated.

According to the pharmacopeial requirements for tablets with average mass above 250 mg, the masses of no more than two tablets may deviate by more than ±5% from the average mass, and no individual tablet may deviate by more than ±10% from the average mass. As for capsules with average mass above 300 mg, the content masses of no more than two capsules may deviate by more than ±7.5% from the average content mass, and no individual capsule content mass may deviate by more than ±15% from the average capsule content mass.

#### 2.2.15. Tablet Hardness, Thickness and Diameter

Tablet hardness was tested on ten randomly selected tablets using a tablet hardness tester (Farmalabor, Canosa di Puglia, Italy). Prior to the hardness test, the diameter and thickness of each individual tablet were measured using a vernier caliper, 24 h after tablet compression.

#### 2.2.16. Content Determination of Ketoprofen from Tablets and Capsules

Ketoprofen content was determined from tablets and capsules, ten samples each. Samples were dissolved in 100 mL of methanol. The samples were exposed to ultrasonic waves for 15 min without heating (Bandelin Sonorex, Berlin, Germany). After tenfold dilution with methanol, ketoprofen content was determined in sample solutions spectrophotometrically (G1103A, Agilent Technologies, Santa Clara, CA, USA) at 260 nm (Section 2.2.18) [26,27].

#### 2.2.17. Dissolution Rate Testing for Tablets and Capsules

The dissolution test was performed using a dissolution apparatus (DT 800 LH, Erweka^®^, Heussenstam, Germany). The test was conducted using a paddle apparatus for tablets and a basket apparatus for capsules at a rotation speed of 50 rpm and a temperature of 37 °C. Two dissolution media were used: pH 1.2 (hydrochloric acid solution) and pH 6.8 (phosphate buffer), both at volume of 900 mL, without maintaining sink conditions. The test was performed in triplicate by sampling 5 mL at 5, 15, 25, 35, 45 and 60 min from the start of the test. Samples were filtered through cellulose acetate membrane filter, 0.45 μm (LLG, Meckenheim, Germany). The concentration of ketoprofen was determined spectrophotometrically (G1103A, Agilent Technologies, Santa Clara, CA, USA) at 260 nm (Section 2.2.18) [26,27].

#### 2.2.18. UV/VIS Spectrophotometry

Ketoprofen content in samples was determined spectrophotometrically (G1103A, Agilent Technologies, Santa Clara, CA, USA) using priorly reported and used method [26,27]. The absorbance was measured at 260 nm. Calibration curves were linear across the range 0.75–40 µg/mL in all three tested media, pH 6.8, pH 1.2 and methanol (R^2^ = 0.9999, R^2^ = 0.9989, R^2^ = 0.9987, respectively).

#### 2.2.19. Statistical Analysis

All numerical data are presented as mean ± standard deviation. Normality of the data was assessed using the Kolmogorov–Smirnov test. Comparisons between groups were performed using one-way analysis of variance (ANOVA) for independent measures, followed by Tukey’s post hoc test to identify statistically significant differences. All statistical analyses were conducted using SPSS software, version 26 (SPSS, Chicago, IL, USA). A *p*-value of less than 0.05 was considered indicative of statistical significance. Dissolution profiles were compared using similarity factor (f_2_). The f_2_ value is calculated using Equation (6):

(6) $$f_{2}=\;50\times \log\left\{{\left(1+\frac{1}{n}\times \sum_{t=1}^{n}{\left(Rt-Tt\right)}^{2}\right)}^{-0.5}\times 100\right\}$$

where *n* is the number of sampling points, Rt is the percentage of drug dissolved from the reference product at time t and Tt is the percentage dissolved from the test product at the same time point. The calculation was performed using mean values of at least three independent dissolution experiments for each formulation, ensuring statistical reliability of the comparison. If f_2_ values were between 50 and 100, the two dissolution profiles were considered similar.

## 3. Results

### 3.1. Appearance of ASDs Prepared by the Fusion Method and HME

**Figure A1:**
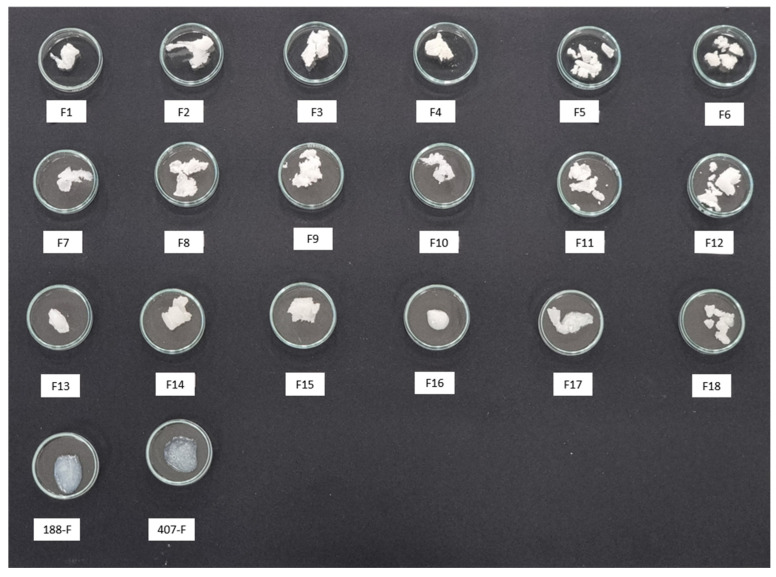
Appearance of the ASDs obtained by the fusion method.

**Figure A2:**
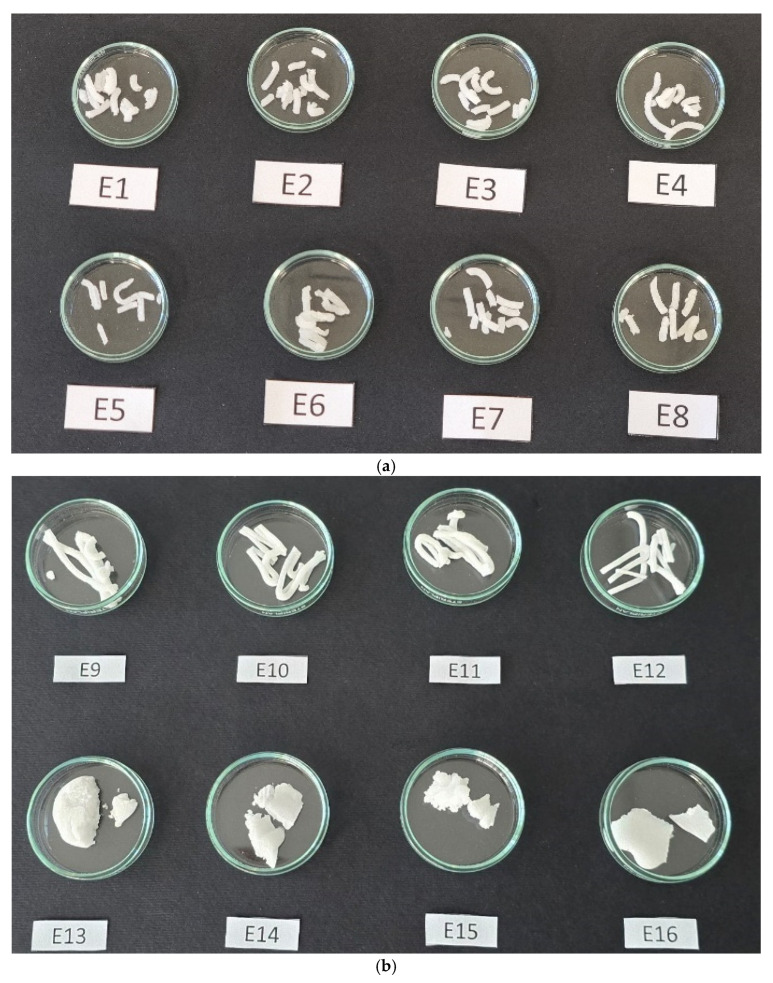
Appearance of the ASDs obtained by HME with (**a**) mannitol and (**b**) PEG 4000 as a hydrophilic carrier.

The appearance of the prepared ASDs is shown in Appendix A (Figure A1 and Figure A2). The ASDs obtained by the fusion method (F1–F18) were all similar in appearance. Those obtained by HME at lower temperatures (E1–E4, E9–E12) were softer and had a rubber-like texture, while those produced at higher temperatures were more brittle and fragile. Re-extrusion caused a slight change in color from white to light beige.

### 3.2. Solubility Study Results for PMs and ASDs Prepared by the Fusion Method

**Figure 1:**
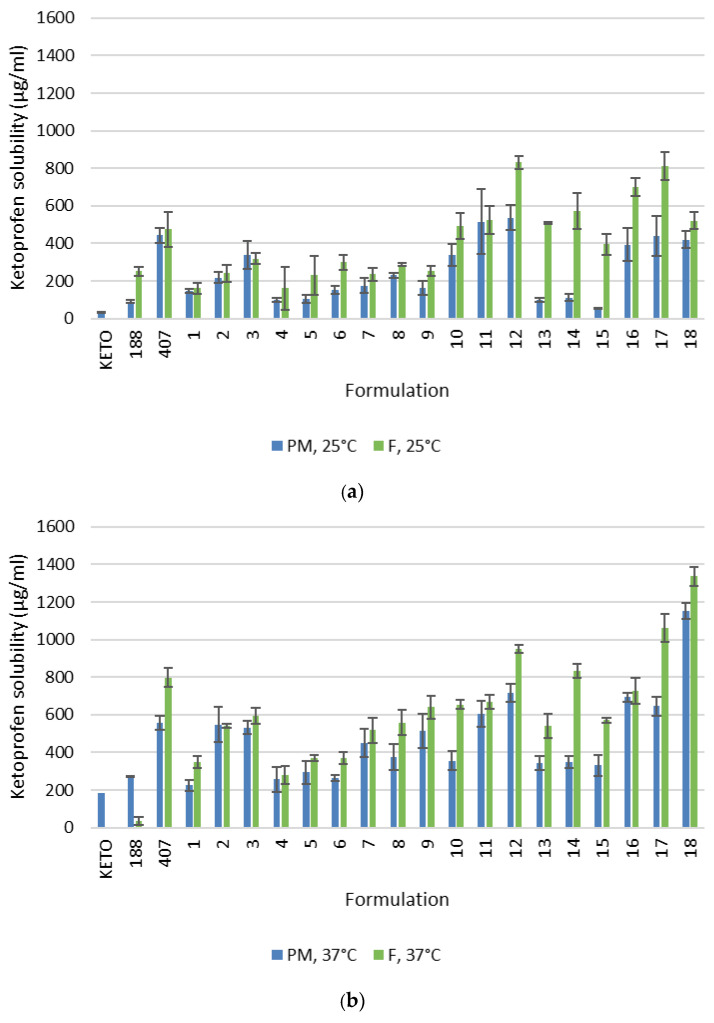
Solubility of ketoprofen alone and from tested PMs and ASDs obtained by the fusion method: (**a**) at 25 °C and (**b**) at 37 °C.

The solubility results for ketoprofen alone, as well as from the tested PMs and ASDs obtained by the fusion method, are shown in Figure 1. In general, ketoprofen showed higher solubility from ASDs compared to the corresponding PMs at both investigated temperatures (25 °C and 37 °C), although some variability between formulations was observed. Among the tested systems, ternary ASDs containing poloxamer 407 tended to provide more pronounced solubility enhancement, with formulations containing higher amounts of hydrophilic carriers performing the best at the biologically relevant temperature of 37 °C. Among the ASDs containing mannitol, F18 demonstrated the highest solubility, while F12 showed the highest solubility among those containing PEG 4000. Overall, formulation F18 exhibited the greatest solubility enhancement at 37 °C. The results of the statistical analysis of the solubility data are presented in Appendix A, Table A1, Table A2, Table A3 and Table A4. At the biorelevant temperature of 37 °C, all ASDs, except F5, showed statistically significant improvements in ketoprofen solubility compared to pure ketoprofen. In addition, among several other ASDs, the best-performing formulations, F12 and F18, exhibited statistically significant increases in ketoprofen solubility compared to their corresponding physical mixtures.

### 3.3. Gibbs Free Energy (∆G°_tr_) Values for PMs and ASDs Prepared by the Fusion Method

The values of Gibbs free energy for PMs and ASDs obtained by the fusion method are presented in Table 4. All ΔG°_tr_ values were negative, confirming favorable solubilization. The ΔG°_tr_ values for the tested PMs and ASDs were generally more negative for ASDs compared to the corresponding PMs at both temperatures (except for PM2 and F2 at 37 °C), indicating enhanced solubilization of ketoprofen from ASDs relative to PMs. Nevertheless, ΔG°_tr_ values were less negative at 37 °C than at 25 °C, suggesting a reduced thermodynamic driving force for solubilization at the higher temperature. As ΔG°_tr_ was calculated based on the solubility of ketoprofen from ASDs relative to that of pure ketoprofen in water (Equation (2)), this difference can be attributed to a more pronounced increase in the solubility of pure ketoprofen with temperature compared to that from the ASDs (Figure 1a,b).

### 3.4. Melting Point Determination Results

The melting points of the pure components and PMs chosen based on solubility results for HME and further testing (PM12 with PEG4000 and poloxamer 407 and PM18 with mannitol and poloxamer 407) are presented in Table 5. The melting points of pure components correlate to the values reported in the literature [30,33]. Formulation PM18 is characterized by a broad melting range, which is slightly lower than the melting range of mannitol, the most dominant component. On the other hand, PM12 showed a similar but slightly higher melting range compared to the carrier PEG 4000.

### 3.5. Solubility Study Results for ASDs Prepared by the HME Method

**Figure 2:**
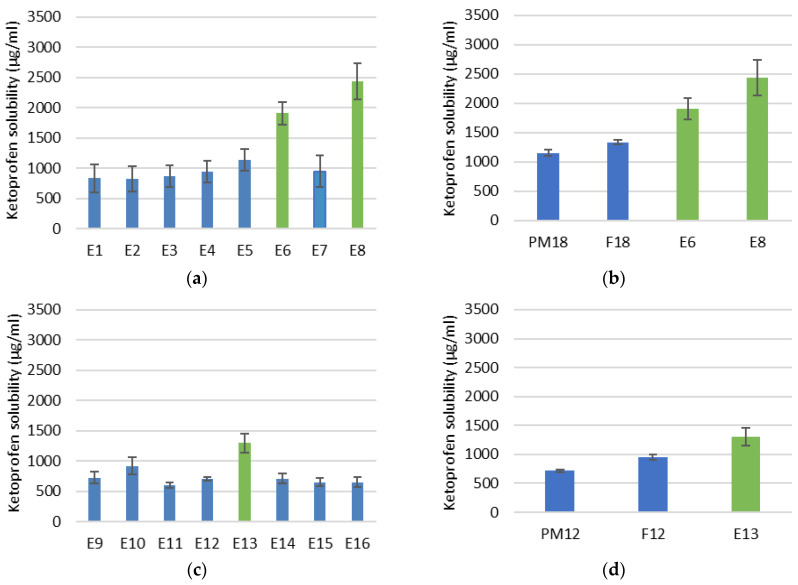
Solubility of ketoprofen from ternary ASDs prepared by HME: (**a**) series with mannitol, (**b**) selected ASD extrudates (E6 and E8) from the mannitol series in comparison with corresponding PM18 and ASD obtained by the fusion method (F18), (**c**) series with PEG 4000 and (**d**) selected ASD extrudate from the PEG 4000 series (E13) in comparison with corresponding PM12 and ASD obtained by the fusion method (F12).

The solubility of ketoprofen from ternary ASDs obtained by the HME method is presented in Figure 2. For formulations containing mannitol as hydrophilic carrier (Figure 2a,b), the greatest increase in solubility was achieved when re-extrusion at elevated temperature was applied (E6 and E8), especially at higher screw speed (E8). On the other hand, for formulations containing PEG 4000 as hydrophilic carrier (Figure 2c,d), the greatest increase in solubility was achieved for E13 obtained at elevated temperature without re-extrusion at a lower screw speed. In line with previous solubility results, a higher increase in ketoprofen solubility was achieved with ternary ASD extrudates containing mannitol.

### 3.6. Gibbs Free Energy (∆G°_tr_) Values for ASDs Prepared by HME

The values of Gibbs free energy for ternary ASDs obtained by the HME method and their comparison with corresponding PMs and ASDs obtained by the fusion method are presented in Table 6. These values correlate well with the solubility results, as the ASDs obtained by HME which exhibited the greatest solubility enhancement also showed the most negative Gibbs free energy values.

### 3.7. Dissolution Results for ASDs Obtained by HME

**Figure 3:**
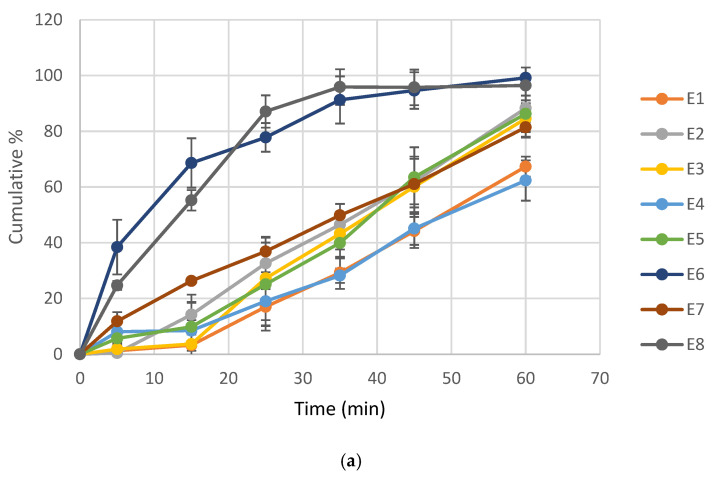

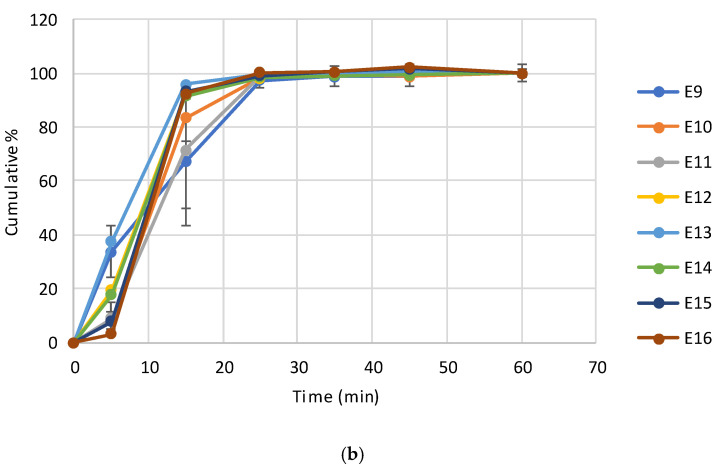
A Dissolution rate of ketoprofen from ternary ASDs obtained by HME with: (**a**) mannitol and (**b**) PEG 4000.

The dissolution rate of ketoprofen from ternary ASDs obtained by HME is shown in Figure 3. The dissolution profiles of ASD extrudates containing mannitol correlate with the solubility data, with formulations E6 and E8 exhibiting accelerated dissolution (Figure 3a). Comparison of dissolution profiles revealed that f_2_ values for E8 and E6 relative to the other formulations were below 50, indicating dissimilar release behavior. In contrast, E6 and E8 displayed similar dissolution profiles (f_2_ = 53.83, Table A5). In contrast, for ASD extrudates containing PEG 4000, despite variability in solubility results, all formulations demonstrated comparable dissolution rates (Figure 3b). Also, f_2_ values were between 50–100 for majority of compared ASD extrudates (Table A6).

### 3.8. FTIR Results

**Figure 4:**
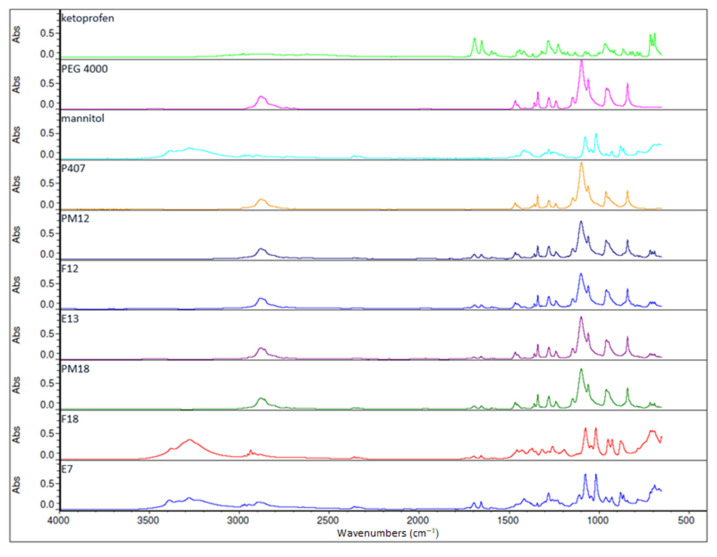
FTIR spectra of pure substances and selected PMs and ASDs.

The FTIR spectra of pure substances and selected PMs and ASDs are presented in Figure 4. The FTIR spectrum of pure ketoprofen exhibited its characteristic absorption bands, including a strong carbonyl stretching peak of the C=O group at 1700 cm^−1^, aromatic C=C vibrations in the region of 1600–1450 cm^−1^, and between 3100 and 3500 cm^−1^ (broad O-H stretching of the carboxylic acid). The spectra of the carriers (PEG 4000, mannitol and poloxamer 407) showed typical C–O–C and C–O stretching bands between 1150 and 1000 cm^−1^, while mannitol additionally displayed a broad O–H stretching region around 3200–3600 cm^−1^. In the physical mixtures (PM12, PM18), all major characteristic peaks of the individual components remained clearly visible without notable shifts, indicating preserved crystallinity and the absence of significant interactions. In contrast, the ASDs obtained by the fusion method (F12, F18) and the HME method (E13, E7) showed a noticeable reduction in the intensity and broadening of ketoprofen’s characteristic carbonyl bands. Specifically, the band at 1700 cm^−1^, corresponding to the carboxylic acid dimer, was significantly diminished in the ASDs, particularly in those prepared via HME. This suggests a disruption of ketoprofen’s crystalline structure and potential molecular-level interactions, such as hydrogen bonding, between the drug and the hydrophilic carriers/surfactants. Overall, the FTIR results support the conclusion that ASDs exhibit decreased crystallinity and enhanced drug–carrier interactions.

### 3.9. DSC Results

**Figure 5:**
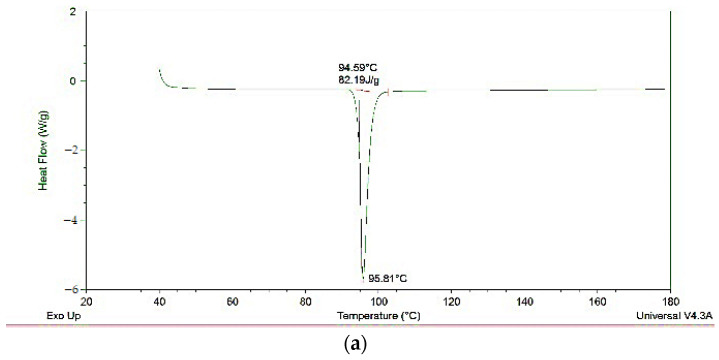

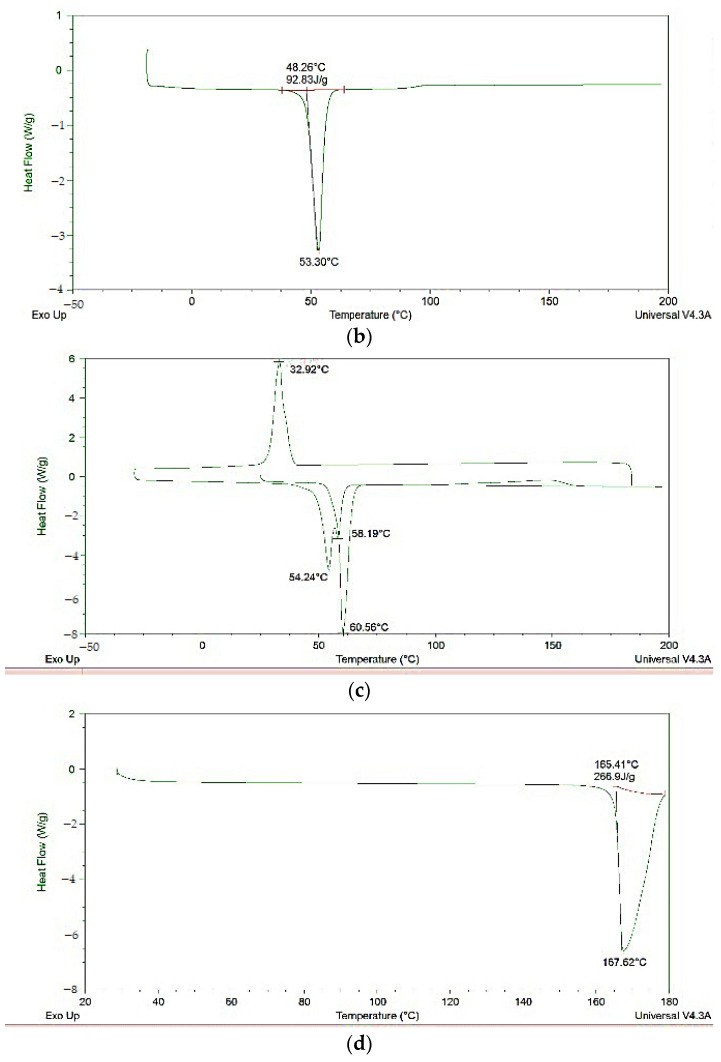
DSC thermograms of pure substances: (**a**) ketoprofen, (**b**) poloxamer 407, (**c**) PEG 4000 and (**d**) mannitol.

**Figure 6:**
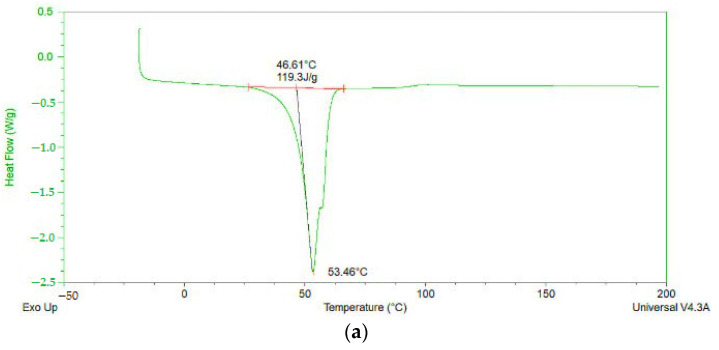

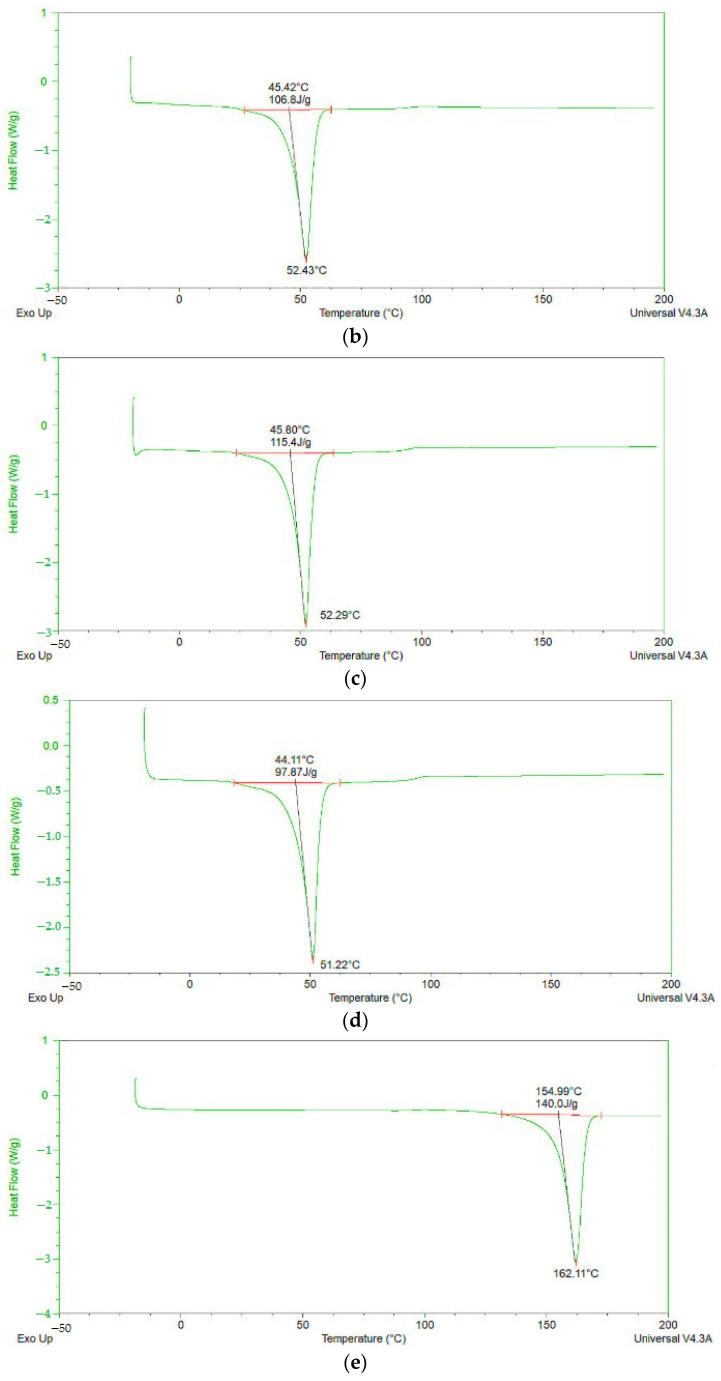

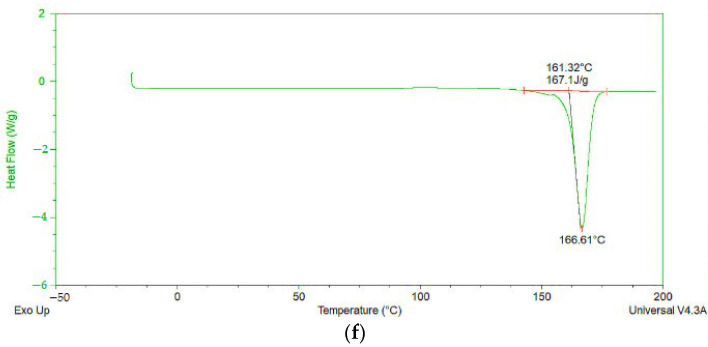
DSC thermograms of tested formulations with PEG 4000: (**a**) PM12, (**b**) F12, (**c**) E13 and with mannitol: (**d**) PM18, (**e**) F18 and (**f**) E7.

The DSC thermograms of pure substances (Figure 5) and selected formulations (Figure 6) provided further evidence of the solid-state changes in the formulations. Pure ketoprofen showed a sharp endothermic melt at 95 °C, consistent with its crystalline nature [34]. In the physical mixtures (PM12, PM18), this melting peak remained well defined, together with the characteristic endotherms of the carriers, indicating that the crystalline nature of the components was preserved. However, the DSC curves for the ASDs, especially those obtained by HME (E7, E13), demonstrated a complete absence of the distinct ketoprofen melting peak. The disappearance or attenuation of the melting peak is a strong indication of amorphization or molecular dispersion of ketoprofen within the polymer matrix. Additionally, the altered thermal behavior of the carriers—manifested as peak shifts and broadening—suggests structural modification due to drug–polymer interactions and thermal–mechanical processing during HME. These DSC findings corroborate the FTIR data and confirm that the extrusion process effectively reduces the crystallinity of ketoprofen, contributing to improved solubility and dissolution behavior.

### 3.10. Flowability Results of Selected ASD Extrudates and Powder Blends for Tablets and Capsules

Ternary ASD extrudates chosen based on both previous solubility results and attributes which could impact further processing (visual appearance, consistency, stickiness) and E7 and E13 obtained by the HME method were subsequently used for preparation of powder blends for tableting (T1 and T2), as well as capsule formulation (K1 and K2). The ASD extrudates and corresponding powder blends were assessed in terms of flowability. The results of the Hausner ratio and compressibility index are presented in Table 7, while the angle of repose data are shown in Table 8. The ASD extrudates exhibited generally fair to good flow properties. Furthermore, the Hausner ratio and compressibility index indicated improved flowability upon incorporation with other excipients in the powder blends.

### 3.11. Uniformity of Mass of Tablets and Capsules

Tablets (T1 and T2) and capsules (K1 and K2), prepared from corresponding powder mixtures, were subsequently assessed for uniformity of mass. The minimum and maximum individual deviations from the average mass were −7.05% and 9.07% for T1, −6.39% and 7.10% for T2, −3.08% and 1.91% for K1 and −3.76% and 2.42% for K2. As more than two tablets deviated by more than ±5% from the average mass in both cases, neither T1 nor T2 fulfilled the Ph. Eur. 11 acceptance criteria [30]. However, the observed deviations were minor and close to the specified acceptance limits; therefore, content uniformity and dissolution studies were nevertheless continued to enable a comprehensive evaluation of the overall performance of the formulations in order to establish a robust basis for subsequent optimization, ultimately supporting the development of final dosage forms with the desired quality attributes. On the other hand, the capsules showed minimal weight variation, and both K1 and K2 fulfilled the Ph. Eur. 11 acceptance criteria for capsules [30].

### 3.12. Diameter, Thickness and Hardness of the Prepared Tablets

**Figure 7:**
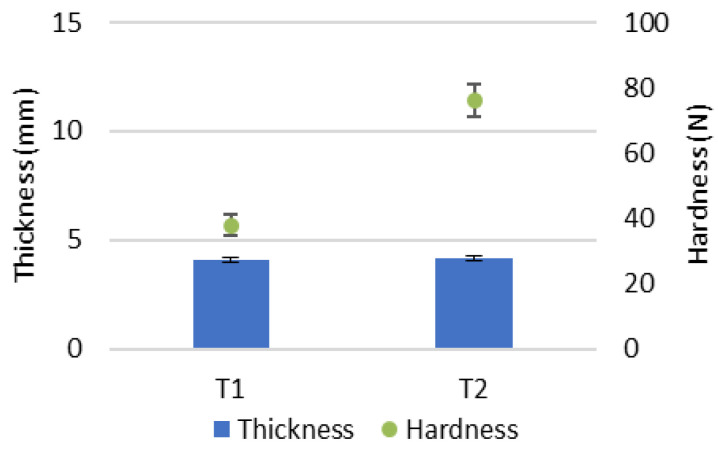
Thickness and hardness of tested tablet formulations.

The prepared tablets (T1 and T2) were assessed in terms of diameter, thickness and hardness. All measured tablet diameters were 12.16 ± 0.01. The results for thickness and hardness measurements are shown in Figure 7. Even though the thickness of tablets T1 and T2 was almost identical, T2 demonstrated greater mechanical strength than T1, as indicated by its higher crushing force.

### 3.13. Content Determination and Dissolution Rate Results for Tablets and Capsules

**Figure 8:**
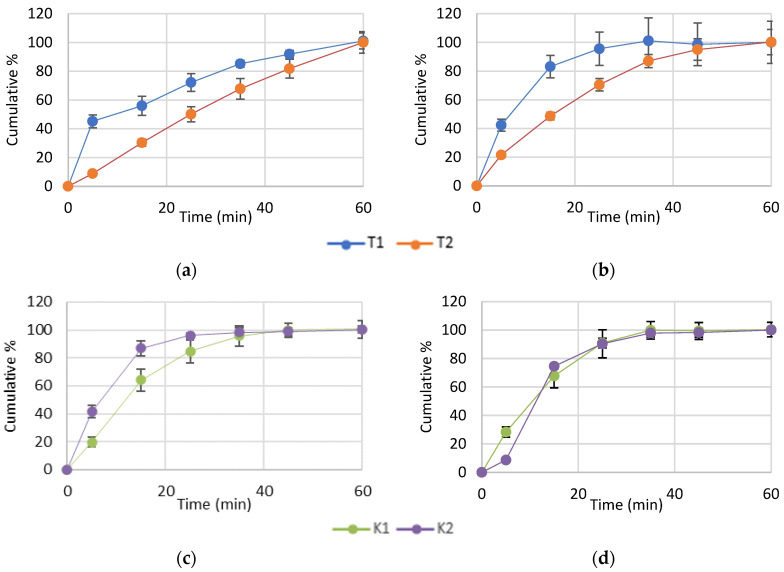
Dissolution profiles of formulations T1 and T2 in the medium: (**a**) pH 1.2, (**b**) pH 6.8 and formulations K1 and K2 in the medium: (**c**) pH 1.2 and (**d**) pH 6.8.

The content of ketoprofen was 98.11 ± 5.74% and 96.71 ± 5.84% in formulations T1 and T2 and 104.82 ± 3.89% and 103.02 ± 2.12% in formulations K1 and K2, which is in line with Ph. Eur. 11 acceptance criteria for Uniformity of content of single-dose preparations [30]. The dissolution rate profiles of tablet formulations (T1 and T2) and capsule formulations (K1 and K2) in pH 1.2 medium and pH 6.8 medium are presented in Figure 8. Since all formulations released more than 80% of the ketoprofen content within 45 min, they meet the Ph. Eur. 11 general acceptance criteria for conventional-release solid oral dosage forms [30]. T1 showed better dissolution rates in both media which is in contrast to the dissolution results obtained for corresponding ASD extrudates, where E7 (corresponding to T1 formulation) showed slow and incomplete dissolution while E13 (corresponding to T2) showed favorable dissolution rates (Figure 3). The dissolution profiles of K1 and K2 were more comparable; however, K2 (corresponding to E13) exhibited slightly better dissolution rates which is more aligned with dissolution data for ASD extrudates (Figure 3). The f_2_ values for the tablet formulations were below 50, indicating dissimilar dissolution profiles in both dissolution media (T1 vs. T2: f_2_ = 34.65 in pH 1.2 and f_2_ = 36.30 in pH 6.8). In contrast, for the capsule formulations, f_2_ indicated dissimilar dissolution profiles in pH 1.2 medium (f_2_ = 44.68), while similar dissolution profiles were observed in pH 6.8 medium (f_2_ = 61.03).

## 4. Discussion

The development of ASDs followed a systematic two-step strategy. Initially, the fusion method was utilized to screen and optimize formulation composition and component ratios. Subsequently, the HME method was employed to systematically investigate the impact of processing parameters on API solubility enhancement and dissolution performance. During handling, more pronounced differences in physical properties were observed among the ASDs prepared by HME. While the ASDs obtained by the fusion method did not exhibit noticeable color changes, slight color alterations were observed in the re-extruded HME samples. The observed discoloration is considered an undesirable effect, likely related to degradation of one or more components of the formulation. This could be further investigated in subsequent studies through readings of the yellowness index and identification of the component(s) responsible for the color change [35]. A similar effect has been reported during investigation of recyclability of poly(lactic acid) (PLA), a biodegradable polymer. When PLA was subjected to repeated extrusions at high temperatures, gradual development of yellowish to reddish hues was documented [35]. In our study, the formulation underwent only two extrusion cycles; therefore, the impact of multiple re-extrusions on ASD color will be investigated in future work. Moreover, imaging of the ASDs using electron microscopy is proposed to fully characterize the degree of ketoprofen homogenization within the carrier matrix [18].

The solubility of PMs and ASDs obtained by the fusion method was examined using a thermostated water bath at 25 °C and 37 °C to evaluate solubility at room and physiological temperature. The solubilities of PM188, PM407 and PM1-18 and their corresponding ASDs (F188, F407, F1-18) demonstrated that the formulation of ASDs increased the solubility of ketoprofen at both tested temperatures, although with variable statistical significance (Figure 1 and Table A1 and Table A2). These results highlight the effectiveness of ASD formation in enhancing API solubility but also demonstrate that the full potential of ASDs can be realized only through appropriate selection and optimization of the components and their ratios [1,4]. Among formulations containing poloxamers, ASDs prepared with poloxamer 407 showed a higher improvement in solubility compared to those containing poloxamer 188. The superior performance of ternary ASDs containing poloxamer 407 compared with those formulated with poloxamer 188 can be attributed to differences in their molecular architecture and micellization behavior. Both poloxamers are triblock copolymers with an EO–PO–EO structure; however, poloxamer 407 possesses a substantially longer hydrophobic poly (propylene oxide) (PO) segment than poloxamer 188, resulting in a higher hydrophobic character and enhanced capacity for drug incorporation. Ketoprofen is a poorly water-soluble BCS Class II drug, and its solubilization is primarily governed by hydrophobic interactions. The larger PO core of poloxamer 407 enables stronger drug–polymer interactions and more efficient accommodation of ketoprofen molecules, both in the solid dispersion and during dissolution. In contrast, the shorter PO block and higher hydrophilicity of poloxamer 188 limit its ability to solubilize and retain hydrophobic drugs. Upon hydration, poloxamer 407 readily forms micellar structures with larger hydrophobic cores and higher aggregation numbers, leading to enhanced micelle-mediated solubilization compared to poloxamer 188, which is consistent with previous reports on poloxamer-based systems [36,37]. Similar solubility improvements were reported in studies of resveratrol, where poloxamer 407 demonstrated a significantly higher increase in both solubility and dissolution rate compared to poloxamer 188 [19]. When the solubilities of ibuproxam formulations with poloxamers 188 and 407 in the form of ASDs were compared, both poloxamers increased solubility, but formulations incorporating poloxamer 407 achieved greater enhancement in solubility and dissolution rate [20]. The advantages of using poloxamer 407 over poloxamer 188 in improving solubility are also evident in studies on nisoldipine ASDs, where poloxamer 407, as part of a ternary ASD formulation, resulted in the fastest release of nisoldipine from tablets [18]. Further comparison of ASDs with the greatest solubility enhancement for each carrier (F18 with ketoprofen, high ratio of mannitol and poloxamer 407 and F12 with ketoprofen, high ratio of PEG 4000 and poloxamer 407) and the corresponding PMs revealed statistically significant increases in ketoprofen solubility from these ASDs relative to the PMs. Based on these results, PM18 and PM12 were chosen for ASD production via the HME method and further investigation.

The Gibbs free energy values were used as indicators of changes in energies and the thermodynamic state of the systems of different formulations at temperatures 25 °C and 37 °C. In the case of PMs, the Gibbs free energy values can additionally provide insight into the degree of miscibility within the system [38]. As shown in Table 4, all PMs and ASDs exhibited negative Gibbs free energy values at both temperatures, indicating thermodynamic stability, good miscibility and favorable solubilization properties. In general, the ASDs showed more negative Gibbs free energy values than their corresponding PMs at both temperatures. An exception was the binary PM2 and F2 at 37 °C, where a slight decrease in negativity of Gibbs free energy value was observed for the ASD compared with the PM, aligning with the solubility results that indicated no advantage of F2 over PM2. Furthermore, since formulation F18 displayed the most negative Gibbs free energy value, this further corroborates the outcomes of the solubility studies. Comparable trends in Gibbs free energy changes have been reported during polymer screening for ASD formulation development, highlighting the usefulness of Gibbs free energy determination in designing ASDs with optimal performance [38]. Nevertheless, the observed less negative Gibbs free energy values at 37 °C compared to 25 °C contradict the solubility results but still reflect the temperature dependence of the solubilization process, since Gibbs free energy is calculated based on the solubility of ketoprofen from ASDs relative to the solubility of pure ketoprofen in water (Equation (2)). Although ketoprofen exhibited higher solubility from ASDs at 37 °C compared to 25 °C, the increase in temperature facilitates disruption of the crystalline lattice of pure ketoprofen to a greater extent, resulting in a more substantial enhancement of the pure API’s aqueous solubility. Consequently, the relative solubility advantage of the ASDs over the pure API is reduced at the higher temperature, leading to less negative ΔG°_tr_ values. The comparatively less pronounced temperature effect on ketoprofen solubility from ASDs may be attributed to the predominantly molecularly dispersed or amorphous nature of the drug in these systems, where solubilization is governed mainly by intermolecular interactions between ketoprofen, solubilization-enhancing additives and water. These interactions have a limited capacity and may be less favorably influenced at elevated temperatures. For example, some polymers and nonionic surfactants can show reduced interactions with hydrophobic drug and water at higher temperatures due to hydrophobic effect and reduced space for drug incorporation [39,40]. This highlights the importance of evaluating solubility enhancement under biorelevant temperature conditions.

The aforementioned results were further supported by melting-point analysis (Table 5). Formulations PM12 (containing PEG 4000 and poloxamer 407) and PM18 (containing mannitol and poloxamer 407) exhibited the most favorable solubility enhancements and Gibbs free energy values, and therefore, the melting points of each individual component, as well as of the final PM formulations, were determined. ASDs are typically characterized by melting temperatures lower than those of their individual constituents [18], a trend that was also observed for PM18. Specifically, the minimum melting point of PM18 was lower than that of poloxamer 407, the component with the lowest melting temperature in the system. Likewise, the maximum melting point of PM18 was lower than that of mannitol, which possesses the highest melting temperature and the widest melting-point range among the formulation components. Consistent with this, PM18 displayed a broader overall melting-point range compared with its individual constituents. On the other hand, PM12 showed a similar but slightly higher melting range compared to PEG 4000 which as carrier represents the proportionally most dominant component of the system.

In the next phase of the research, ketoprofen ASDs were prepared by HME to evaluate the impact of extrusion on the solubility of formulations F12 (containing PEG 4000 and poloxamer 407) and F18 (containing mannitol and poloxamer 407). The investigated processing parameters included temperature and screw speed, both of which have been shown to influence intermolecular mixing and extrudability [41,42,43,44], as well as the number of extrusion cycles, since re-extrusion is expected to further reduce particle size and enhance dispersion of the API within the carrier matrix. Processing temperatures were selected based on the melting points of the physical mixtures and pure API, taking into account the literature-reported thermal stability of all components [21,22,23,24,25]. Screw speed was varied in the mid-range to ensure effective mixing while maintaining sufficient residence time during extrusion. The same powder blends were processed under varying extrusion conditions (Table 2), and the resulting solubility data (Figure 2) and Gibbs free energy values (Table 6) indicated an improvement in solubility when processing was performed at 160 °C in the first heating zone and 165 °C in the second. For formulations with mannitol, a particularly pronounced solubility enhancement and the most negative values of the Gibbs free energy were observed for E6 and E8, which underwent re-extrusion under the same temperature conditions. Among them, extrudate E8, produced at higher screw speed, exhibited the greatest solubility increase. However, this effect may be carrier-dependent, as ASD extrudates containing PEG 4000 did not exhibit improved solubility upon re-extrusion. In contrast, formulation E13, which was not re-extruded and was produced at a lower screw speed, demonstrated the highest solubility and most negative values of Gibbs free energy.

The Gibbs free energy values for ASDs obtained by HME correlate well with the solubility results, as the ASD extrudates which exhibited the greatest solubility enhancement also showed the most negative Gibbs free energy values, more negative than the corresponding PMs and ASDs obtained by fusion method. The findings indicate that extrusion generally increased the solubility of the systems, most likely as a result of improved molecular ordering, i.e., enhanced mixing of ketoprofen with the hydrophilic carrier [38]. These results further demonstrated the significance of determining Gibbs free energy with the aim of selecting appropriate processing parameters for HME so that the system after formulation would be homogeneous and thermally stable [38].

An increase in processing temperature and screw speed has been reported to enhance API solubility and dissolution from ASDs. Elevated processing temperatures can facilitate disruption of the crystalline lattice of the API and promote drug–carrier intermolecular interactions, while both higher temperature and increased screw speed reduce melt viscosity, thereby improving mixing efficiency and extrudability [41,42,43,44]. In the present study, higher processing temperatures led to enhanced API solubility for ASD extrudates prepared with both hydrophilic carriers. In contrast, at favored higher processing temperatures, the effect of screw speed was carrier-dependent: higher screw speed was beneficial for ASD extrudates containing mannitol, whereas lower screw speed resulted in improved performance for ASD extrudates prepared with PEG 4000. In the case of PEG 4000, increased shear stress may have induced polymer chain scission, which can compromise the stabilization of the amorphous drug and promote API recrystallization [43]. Re-extrusion was investigated with the expectation that an additional extrusion cycle would further reduce API particle size and improve its dispersion within the carrier matrix, thereby enhancing solubility and dissolution. API solubility from ASD extrudates containing mannitol indeed increased when produced by re-extrusion. While previous studies on re-extrusion have primarily focused on the recycling of biodegradable extrudates [35], our findings suggest that re-extrusion may also be beneficial in the development of ASD extrudates containing thermally stable compounds. In contrast, no improvement was observed for PEG 4000-based ASD extrudates, which is consistent with the poorer performance of PEG 4000 under shear stress during single-pass extrusion. Moreover, the appearance of discoloration indicates possible degradation in case of both ASD formulations, highlighting one of the major drawbacks associated with high processing temperatures and shear stress [41,43].

The same ketoprofen ASD extrudates were also evaluated for dissolution rate to further investigate the release profile of ketoprofen (Figure 3). The results demonstrated that the potential of the ASD extrudates to release ketoprofen more quickly from the formulation is in line with the solubility data. Similar trends have been reported for ketoprofen ASDs prepared by the fusion method, suggesting that, regardless of the preparation method, the formulation itself is the key driving factor for increased solubility and dissolution rate [45]. Among the extruded ASDs with mannitol, E6 and E8 exhibited higher ketoprofen release rates, with notable differences observed as early as the first measurement at 5 min, a trend that persisted throughout the experiment and was consistent with the solubility results. Complete drug release from E6 and E8 was achieved by 35 min, whereas for ASD extrudates E1, E2, E3, E4, E5 and E7, lower ketoprofen concentrations were measured at the same time point. Accordingly, the influence of process parameters on dissolution in mannitol-based formulations followed the same trends as those observed for solubility. Contrary to ASD extrudates with mannitol, dissolution profiles of ASD extrudates with PEG 4000 were generally more favorable and comparable, all of them achieving complete release at the 25-min mark, indicating that no discernible effect of process parameters on dissolution could be identified. The more pronounced enhancement of API dissolution rate compared to API solubility from ASD extrudates with PEG 4000 may be attributed to formulation-related factors, such as improved surface wetting and solubilization of the API at the ASD–dissolution medium interface by PEG and poloxamer 407 and creation of a concentration gradient between the diffusion layer and the bulk solution, thereby facilitating dissolution even from extrudates which did not show great improvements in API solubility. In addition, the higher dissolution rate of E13 containing PEG 4000 compared to E6 and E8 formulated with mannitol is consistent with a previous report in the literature where PEG 4000 also showed higher increase in API dissolution compared to mannitol [16]. This could be explained by the reported tendency of API to recrystallize from mannitol [4,16]. Overall, given that ketoprofen is an analgesic for which rapid release is desirable, these results highlight the strong potential of the ASD formulation for further development and application.

The FTIR (Figure 4) and DSC (Figure 5 and Figure 6) results mutually confirm the successful formation of ASDs and provide insight into the underlying mechanism of solubility enhancement. The FTIR spectral changes, particularly the attenuation of the carboxylic acid dimer peak of ketoprofen in the ASDs, indicate a disruption of the drug’s crystal lattice. This phenomenon is likely due to the formation of intermolecular interactions, such as hydrogen bonds, between the carboxylic acid group of ketoprofen and the hydroxyl groups of mannitol/poloxamer or the ether oxygen atoms of PEG/poloxamer. The more pronounced effect observed in the HME-processed samples emphasizes the superior mixing and intimate contact achieved through the extrusion process, leading to a more complete amorphization and molecular dispersion of the API. The DSC findings support the FTIR data. The disappearance of the sharp ketoprofen melting endotherm in the HME formulations is an indicator of amorphization. When a drug is converted to its amorphous form, the long-range order of the crystal lattice is lost, which reduces or leads to the disappearance of melting peaks. This amorphous state is thermodynamically metastable and possesses higher free energy, leading to a higher apparent solubility. These more pronounced effects observed by FTIR and DSC for HME-produced ASDs are in line with the enhanced solubility results for these ASD extrudates compared to PMs and ASDs obtained by the fusion method (Figure 2b,d).

From the ASDs produced via HME, formulations E7 (from mannitol series) and E13 (from PEG 4000 series) were selected for incorporation into powder blends intended for tablet and capsule preparation. The selection criteria were based not only on the previous solubility results but also on the attributes of the ASD extrudates that could impact further processing (visual appearance, consistency and stickiness). Although E6 and E8 exhibited superior solubility and dissolution performance, both formulations showed discoloration, unfavorable consistency and stickiness resulting from re-extrusion. Therefore, E7 (high processing temperature, high screw speed, single extrusion) was chosen from the mannitol series, as it demonstrated more favorable properties together with the best dissolution profile among the non–re-extruded ASD extrudates from this series. The powder blends used for tablet and capsule preparation exhibited good flowability, which is essential for tableting and capsule filling to ensure uniform mass and the production of tablets and capsules with consistent and reproducible properties. The resulting tablets had uniform diameter and thickness; however, they displayed unfavorable variability in individual mass measurements, which could be improved in the future through appropriate formulation adjustments to optimize flowability and compression behavior. Nevertheless, both dosage forms demonstrated uniform ketoprofen content. The capsules showed little weight variation and more favorable dissolution profiles compared with the tablets, highlighting the influence of excipient composition and the direct-compression process on formulation characteristics and dissolution properties [46,47]. In particular, the dense particle packing associated with direct compression, together with the well-documented tendency of magnesium stearate to form hydrophobic surface coatings that inhibit wetting, disintegration and dissolution, especially under acidic conditions [48], may account for the slower dissolution observed for the tablet formulations compared with the capsules. The better dissolution profile of T1 compared to T2 could indicate more pronounced interaction between magnesium stearate with PEG 4000 compared to mannitol.

## 5. Conclusions

The combination of ketoprofen with mannitol or PEG 4000 was effective in formulating amorphous solid dispersions (ASDs) with enhanced solubility by both the fusion method and the HME method. The addition of poloxamer 188 and 407 further increased the solubility, with the effect being more pronounced in formulations containing poloxamer 407, attributed to its longer hydrophobic core. Differences in Gibbs free energy trends at 25 °C and 37 °C underscored the importance of performing experiments under biorelevant conditions. The formulations that proved best in increasing solubility and dissolution rates at body temperature were those with high-concentration hydrophilic carrier (mannitol or PEG 4000) and poloxamer 407 as the surfactant. Among HME processing parameters, temperature was identified as the most critical factor, with higher temperatures consistently yielding ASD extrudates with superior solubility. In contrast, the effects of screw speed and re-extrusion were carrier-dependent, showing positive effects for mannitol-based ASD extrudates but not for PEG 4000. Re-extrusion was found to be a significant extrusion parameter for increasing the solubility of ketoprofen from the tested ASD extrudates with mannitol; however, it let to discoloration, unfavorable consistency and stickiness which need to be further investigated. The structural (FTIR) and thermal (DSC) analyses confirmed that the enhanced solubility and dissolution performance of the ternary ASDs, particularly those prepared via HME, are a direct consequence of the successful transformation of ketoprofen into an amorphous state and its molecular-level interaction with the hydrophilic carriers and surfactants, forming a stable, single-phase system. The selected HME-produced ASDs were successfully formulated into powder blends with good flowability, resulting in tablets and capsules with good physical characteristics and dissolution profiles, which could be further enhanced through future formulation optimization.

## Figures and Tables

**Table 1:** Composition (mass ratio) of prepared PMs and ASDs obtained by the fusion method.

Formulation		Ketoprofen	PEG 4000	Mannitol	Poloxamer 188	Poloxamer 407
PM188	F188	1			1	
PM407	F407	1				1
PM1	F1	1	1			
PM2	F2	1	3			
PM3	F3	1	5			
PM4	F4	1		1		
PM5	F5	1		3		
PM6	F6	1		5		
PM7	F7	1	1		1	
PM8	F8	1	3		1	
PM9	F9	1	5		1	
PM10	F10	1	1			1
PM11	F11	1	3			1
PM12	F12	1	5			1
PM13	F13	1		1	1	
PM14	F14	1		3	1	
PM15	F15	1		5	1	
PM16	F16	1		1		1
PM17	F17	1		3		1
PM18	F18	1		5		1

**Table 2:** HME processing parameters applied for ASD preparation.

Formulation	Temperature (°C)		Screw Speed (rpm)	Number of Extrusions
	Heater 1	Heater 2		
E1	130	135	30	1
E2	130	135	30	2
E3	130	135	50	1
E4	130	135	50	2
E5	160	165	30	1
E6	160	165	30	2
E7	160	165	50	1
E8	160	165	50	2
E9	130	135	30	1
E10	130	135	30	2
E11	130	135	50	1
E12	130	135	50	2
E13	160	165	30	1
E14	160	165	30	2
E15	160	165	50	1
E16	160	165	50	2

**Table 3:** Composition of tablet and capsule blend formulations.

Formulation	Lactose [g]		Sodium Starch Glycolate [g]	Magnesium Stearate [g]	ASDs Containing Ketoprofen [g]
	Super Tab 21AN	Capsulac^®^			
T1/T2 ^1^	14.875 ^3^	/	1.25	0.125	8.75
K1/K2 ^2^	/	20.4 [mL] ^4^	/	/	5.25

^1^ T1—tablet formulation with mannitol and T2—tablet formulation with PEG 4000. ^2^ K1—capsule formulation with mannitol and K2—capsule formulation with PEG 4000. ^3^ Calculation for mass of 50 tablets (predicted mass of one tablet 0.5 g) ^4^ Calculation for mass of 30 capsules (size 0, volume of one capsule 0.68 mL).

**Table 4:** Values of Gibbs free energy ΔG°_tr_ for PMs and ASDs obtained by the fusion method.

Formulation	∆G°_tr_ (Jmol^−1^) at 25 °C	∆G°_tr_ (Jmol^−1^) at 37 °C	Formulation	∆G°_tr_ (Jmol^−1^) at 25 °C	∆G°_tr_ (Jmol^−1^) at 37 °C
PM188	−2129.18	−1044.50	F188	−4706.77	−1813.94
PM407	−6097.08	−3071.06	F407	−6273.17	−3582.98
PM1	−3429.26	−362.66	F1	−3617.51	−1676.40
PM2	−4353.02	−2831.75	F2	−4592.50	−2807.36
PM3	−4037.96	−2642.66	F3	−5424.98	−3149.97
PM4	−2417.99	−875.08	F4	−3595.37	−1098.62
PM5	−2485.06	−1313.66	F5	−5378.56	−1831.59
PM6	−3739.83	−951.13	F6	−5134.07	−1837.17
PM7	−3794.73	−2332.97	F7	−4529.03	−2866.99
PM8	−4490.80	−1969.92	F8	−5114.11	−3117.39
PM9	−3604.90	−2825.75	F9	−4705.99	−3238.14
PM10	−5414.65	−1960.11	F10	−6488.89	−3308.04
PM11	−6479.55	−3162.55	F11	−6769.28	−3347.22
PM12	−6359.29	−3553.53	F12	−7542.36	−4265.71
PM13	−2255.94	−1625.02	F13	−6466.14	−2795.30
PM14	−2669.20	−1674.33	F14	−6857.09	−3899.55
PM15	−1286.32	−1546.47	F15	−5726.39	−2899.63
PM16	−6260.00	−3321.68	F16	−6957.63	−3566.40
PM17	−6973.31	−3387.27	F17	−7589.03	−4433.72
PM18	−5335.56	−4759.67	F18	−6499.05	−5071.99

**Table 5:** Melting point determination results.

Substance/Mixture	Melting Point (°C)	
	Min	Max
Ketoprofen	93.2	95
PEG 4000	53.5	59.1
Mannitol	66.5	167.9
Poloxamer 407	53.2	56.4
PM12	53.9	63.1
PM18	42.2	165.2

**Table 6:** Values of Gibbs free energy for ternary ASDs obtained by the HME method (E1–E16) and their comparison with corresponding PMs and ASDs obtained by the fusion method.

Formulation	∆G°_tr_ (Jmol^−1^) at 37 °C	Formulation	∆G°_tr_ (Jmol^−1^) at 37 °C
PM18	−4759.67	PM12	−3553.53
F18	−5071.99	F12	−4265.71
E1	−3927.12	E9	−3556.80
E2	−3898.11	E10	−4177.13
E3	−4022.77	E11	−3074.71
E4	−4234.62	E12	−3489.86
E5	−4723.57	E13	−5065.42
E6	−6056.73	E14	−3514.54
E7	−4260.28	E15	−3289.77
E8	−6685.64	E16	−3287.44

**Table 7:** Hausner ratio and compressibility index of extrudates E7 and E13 and powder blends (T1, T2, K1 and K2).

Formulation	Hausner Ratio	Compressibility Index	Degree of Flowability ^1^
E7	1.14 ± 0.03	12.07 ± 2.22	Good
T1	1.07 ± 0.00	6.13 ± 0.21	Excellent
K1	1.10 ± 0.04	9.06 ± 3.05	Excellent
E13	1.21 ± 0.05	17.61 ± 3.21	Fair
T2	1.13 ± 0.00	11.33 ± 0.38	Good
K2	1.06 ± 0.00	5.46 ± 0.17	Excellent

^1^ The degree of flowability was assessed in line with Ph. Eur. 11 requirements [30].

**Table 8:** Angle of repose of extrudates E7 and E13 and powder blends (T1, T2, K1 and K2).

Formulation	Angle of Repose	Degree of Flowability ^1^
E7	35.63 ± 0.69	Fair
T1	34.61 ± 0.92	Good
K1	35.87 ± 0.75	Fair
E13	37.90 ± 0.52	Fair
T2	39.61 ± 0.33	Fair
K2	38.49 ± 1.17	Fair

^1^ The degree of flowability was assessed in line with Ph. Eur. 11 requirements [30].

## Data Availability

The datasets generated during and/or analyzed during the current study are available from the corresponding author on reasonable request.

## Abbreviations

The following abbreviations are used in this manuscript:

API	active pharmaceutical ingredient
ASD	amorphous solid dispersion
PEG	polyethylene glycol
HME	hot melt extrusion
BCS	Biopharmaceutics Classification System
PM	physical mixture
FTIR	Fourier Transform Infrared
DSC	Differential Scanning Calorimetry
PO	poly(propylene oxide)
PLA	poly(lactic acid)

## Appendix A

**Table A1:** ANOVA statistics for the solubility data of pure ketoprofen, PMs and ASDs obtained by the fusion method at 25 °C.

	Keto	PM188	PM407	PM1	PM2	PM3	PM4	PM5	PM6	PM7	PM8	PM9	PM10	PM11	PM12	PM13	PM14	PM15	PM16	PM17	PM18
keto		0.999	0.001	0.997	0.425	0.001	0.999	0.999	0.993	0.919	0.302	0.975	0.001	0.001	0.001	0.999	0.999	0.999	0.001	0.001	0.001
F188	0.124	0.701																			
F407	0.001		0.999																		
F1	0.977			0.999																	
F2	0.198				0.999																
F3	0.003					0.999															
F4	0.976						0.999														
F5	0.002							0.999													
F6	0.010								0.852												
F7	0.250									0.999											
F8	0.020										0.999										
F9	0.123											0.999									
F10	0.001												0.801								
F11	0.001													0.999							
F12	0.001														0.002						
F13	0.001															0.001					
F14	0.001																0.001				
F15	0.001																	0.001			
F16	0.001																		0.001		
F17	0.001																			0.001	
F18	0.001																				0.999

**Table A2:** ANOVA statistics for the solubility data of pure ketoprofen, PMs and ASDs obtained by the fusion method at 37 °C.

	Keto	PM188	PM407	PM1	PM2	PM3	PM4	PM5	PM6	PM7	PM8	PM9	PM10	PM11	PM12	PM13	PM14	PM15	PM16	PM17	PM18
keto		0.968	0.001	0.999	0.001	0.001	0.999	0.760	0.994	0.001	0.005	0.001	0.028	0.001	0.001	0.078	0.049	0.151	0.001	0.001	0.001
F188	0.177	0.001																			
F407	0.001		0.001																		
F1	0.001			0.503																	
F2	0.048				0.999																
F3	0.001					0.999															
F4	0.001						0.999														
F5	0.934							0.997													
F6	0.009								0.806												
F7	0.008									0.999											
F8	0.001										0.017										
F9	0.001											0.454									
F10	0.001												0.001								
F11	0.001													0.999							
F12	0.001														0.001						
F13	0.001															0.004					
F14	0.001																0.001				
F15	0.001																	0.001			
F16	0.001																		0.999		
F17	0.001																			0.001	
F18	0.001																				0.015

**Table A3:** ANOVA statistics for the solubility data among selected ASDs obtained by the fusion method with different ratios of PEG 4000 as a hydrophilic carrier.

**ANOVA (Solubility at 25** **°** **C)**			
	F10	F11	F12
F10		0.999	0.001
F11	0.999		0.001
F12	0.001	0.001	
**ANOVA (Solubility at 37** **°** **C)**			
	F10	F11	F12
F10		0.999	0.001
F11	0.999		0.001
F12	0.001	0.001	

**Table A4:** ANOVA statistics for the solubility data among selected ASDs obtained by the fusion method with different ratios of mannitol as a hydrophilic carrier.

**ANOVA (Solubility at 25** **°** **C)**			
	F16	F17	F18
F16		0.996	0.497
F17	0.996		0.002
F18	0.497	0.002	
**ANOVA (Solubility at 37** **°** **C)**			
	F16	F17	F18
F16		0.001	0.001
F17	0.001		0.001
F18	0.001	0.001	

**Table A5:** Comparison of dissolution profiles of ASD extrudates with mannitol (values of factor of similarity, f_2_ *).

Factor of Similarity (f_2_)							
	E2	E3	E4	E5	E6	E7	E8
E1	42.32	47.81	69.89	50.32	15.52	38.86	15.95
E2		64.96	41.21	63.00	21.51	57.20	22.39
E3			45.28	72.36	19.29	49.00	20.13
E4				49.23	16.17	40.08	16.43
E5					19.62	52.52	20.16
E6						24.49	53.83
E7							24.88

* f_2_ value between 50 and 100 indicates that the two dissolution profiles are similar.

**Table A6:** Comparison of dissolution profiles of ASD extrudates with PEG4000 (values of factor of similarity, f_2_ *).

Factor of Similarity (f_2_)							
	E10	E11	E12	E13	E14	E15	E16
E9	47.17	50.98	48.23	47.88	47.69	42.66	41.05
E10		66.96	63.57	46.06	65.73	69.28	68.01
E11			53.09	42.48	53.92	53.92	54.07
E12				57.55	96.06	66.74	59.77
E13					55.72	47.44	44.21
E14						69.64	61.94
E15							83.51

* f_2_ value between 50 and 100 indicates that the two dissolution profiles are similar.

## References

[1] Cid A.G., Simonazzi A., Palma S.D., Bermudez J.M. (2019). Solid dispersion technology as a strategy to improve the bioavailability of poorly soluble drugs. Ther. Deliv..

[2] Oliveira V.D.S., de Almeida A.S., Albuquerque I.D.S., Duarte F.I.C., Queiroz B.C.S.H., Converti A., Lima A.A.N. (2020). Therapeutic Applications of Solid Dispersions for Drugs and New Molecules: In Vitro and In Vivo Activities. Pharmaceutics.

[3] Newman A., Knipp G., Zografi G. (2012). Assessing the performance of amorphous solid dispersions. J. Pharm. Sci..

[4] Malkawi R., Malkawi W.I., Al-Mahmoud Y., Tawalbeh J. (2022). Current Trends on Solid Dispersions: Past, Present, and Future. Adv. Pharmacol. Pharm. Sci..

[5] Bhatia M., Devi S. (2020). Development, characterisation and evaluation of PVP K-30/PEG solid dispersion containing ketoprofen. Acta Pharm. Sci..

[6] Chen W., Ouyang D. (2017). Investigation of molecular dissolution mechanism of ketoprofen binary and ternary solid dispersions by molecular dynamics simulations. Mol. Simul..

[7] Han R., Huang T., Liu X., Yin X., Li H., Lu J., Ji Y., Sun H., Ouyang D. (2019). Insight into the Dissolution Molecular Mechanism of Ternary Solid Dispersions by Combined Experiments and Molecular Simulations. AAPS PharmSciTech.

[8] Browne E., Worku Z.A., Healy A.M. (2020). Physicochemical Properties of Poly-Vinyl Polymers and Their Influence on Ketoprofen Amorphous Solid Dispersion Performance: A Polymer Selection Case Study. Pharmaceutics.

[9] Mura P., Moyano J.R., González-Rodríguez M.L., Rabasco-Alvaréz A.M., Cirri M., Maestrelli F. (2005). Characterization and dissolution properties of ketoprofen in binary and ternary solid dispersions with polyethylene glycol and surfactants. Drug. Dev. Ind. Pharm..

[10] Patel D., Patel S., Patel C. (2014). Formulation and evaluation of fast dissolving tablet containing domperidone ternary solid dispersion. Int. J. Pharm. Investig..

[11] Vijaya Laxmi M., Naik S., Yadav S., Vijaya Charan G. (2025). Effect of Surfactants on Lansoprazole Solid Dispersions: A Pathway to Improved Dissolution and Development of Fast Disintegrating Tablets. J. Neonatal Surg..

[12] Alshetaili A., Alshahrani S.M., Almutairy B.K., Repka M.A. (2020). Hot Melt Extrusion Processing Parameters Optimization. Processes.

[13] Demuth B., Nagy Z.K., Balogh A., Vigh T., Marosi G., Verreck G., Van Assche I., Brewster M.E. (2015). Downstream processing of polymer-based amorphous solid dispersions to generate tablet formulations. Int. J. Pharm..

[14] Dalvi P.B., Gerange A.B., Ingale P.R. (2015). Solid dispersion: Strategy to enhance solubility. J. Drug Delivery Ther..

[15] Shohin I.E., Kulinich J.I., Ramenskaya G.V., Abrahamsson B., Kopp S., Langguth P., Polli J.E., Shah V.P., Groot D.W., Barends D.M. (2012). Biowaiver monographs for immediate-release solid oral dosage forms: Ketoprofen. J. Pharm. Sci..

[16] Palanisamy M., Khanam J. (2011). Solid dispersion of prednisolone: Solid state characterization and improvement of dissolution profile. Drug Dev. Ind. Pharm..

[17] Poka M.S., Milne M., Wessels A., Aucamp M. (2023). Sugars and Polyols of Natural Origin as Carriers for Solubility and Dissolution Enhancement. Pharmaceutics.

[18] Manimaran V., Damodharan N. (2016). Development of Fast Dissolving Tablets of Nisoldipine by Solid Dispersion Technology using Poloxamer 407 and Poloxamer 188. J. Young Pharm..

[19] Vasconcelos T., Prezotti F., Araújo F., Lopes C., Loureiro A., Marques S., Sarmento B. (2021). Third-generation solid dispersion combining Soluplus and poloxamer 407 enhances the oral bioavailability of resveratrol. Int. J. Pharm..

[20] Cirri M., Mura P., Rabasco A.M., Ginés J.M., Moyano J.R., Gònzalez-Rodrìguez M.L. (2004). Characterization of ibuproxam binary and ternary dispersions with hydrophilic carriers. Drug Dev. Ind. Pharm..

[21] Alshetaili A.S., Almutairy B.K., Tiwari R.V., Morott J.T., Alshehri S.M., Feng X., Alsulays B.B., Park J.B., Zhang F., Repka M.A. (2016). Preparation and Evaluation of Hot-Melt Extruded Patient-Centric Ketoprofen Mini-Tablets. Curr. Drug. Deliv..

[22] GungorErtugral T., Coskun Y., Oral A., Ulusoy S. (2024). Preparation and Characterization of Poly(lactic acid)-Based Poly(ethylene glycol) and Daphne Essential Oil-Loaded Smart Nanofibers for Thermal Protection. ACS Omega.

[23] Kumaresan G., Velraj R., Iniyan S. (2011). Thermal Analysis of D-mannitol for Use as Phase Change Material for Latent Heat Storage. J. Appl. Sci..

[24] Nanaki S., Eleftheriou R.M., Barmpalexis P., Kostoglou M., Karavas E., Bikiaris D. (2019). Evaluation of Dissolution Enhancement of Aprepitant Drug in Ternary Pharmaceutical Solid Dispersions with Soluplus^®^ and Poloxamer 188 Prepared by Melt Mixing. Sci.

[25] Ly H.Q., Chen Y.J., Nguyen V.T., Tseng C.L. (2025). Optimization of the poloxamer 407-conjugated gelatin to synthesize pH-sensitive nanocarriers for controlled paclitaxel delivery. J. Polym. Res..

[26] Kormosh Z., Hunka I., Basel Y. (2009). Spectrophotometric determination of ketoprofen and its application in pharmaceutical analysis. Acta Pol. Pharm..

[27] Gavat C.C. (2023). Ultraviolet (UV) Spectrophotometric Analysis of Ketoprofen in Tablets–Statistical Validation of Proposed Method. Mater. Proc..

[28] Poša M. (2025). Theoretical View: Thermodynamics of the Saturation Dissolution of a Molecular (Solid) Dispersion of a Hydrophobic Molecule and Polymeric Surfactant in an Aqueous Solution. Int. J. Mol. Sci..

[29] Gavarić N., Todorović N., Popović S., Božić I., Vojnović A., Milošević N., Lalić-Popović M. (2025). Spectroscopic Analysis for the Characterization of 3D-Printed Zinc Supplements for Tailored Veterinary Treatment. Chemosensors.

[30] (2023). European Pharmacopoeia.

[31] Todorović N.B., Ćoškov A.D., Čanji Panić J.M., Bajić D.D., Švonja Parezanović G.D., Milošević N.P., Lalić-Popović M.N. (2025). Paradoxical Effects of Superdisintegrants on Dissolution Performance in Ibuprofen Orodispersible Tablets. Dissolution Technol..

[32] Todorović N.B., Sazdanić D.B., Mamužić B.G., Lalić-Popović M.N., Atanacković Krstonošić M.T., Mikulić M.P. (2025). Chemical Stability and Dissolution of Paracetamol Tablets Formulated by Direct Compression and Granulation Process. Acta Chim. Slov..

[33] Rowe R.C., Sheskey P.J., Quinn M.E. (2009). Handbook of Pharmaceutical Excipients.

[34] Reda R.I., Wen M.M., El-Kamel A.H. (2017). Ketoprofen-loaded Eudragit electrospun nanofibers for the treatment of oral mucositis. Int. J. Nanomed..

[35] Gonçalves L.M.G., Rigolin T.R., Frenhe B.M., Bettini S.H.P. (2020). On the Recycling of a Biodegradable Polymer: Multiple Extrusion of Poly (Lactic Acid). Mater. Res..

[36] Alexandridis P., Hatton T.A. (1995). Poly(ethylene oxide)–poly(propylene oxide)–poly(ethylene oxide) block copolymer surfactants in aqueous solutions and at interfaces. Colloids Surf. A.

[37] Kabanov A.V., Alakhov V.Y. (2002). Pluronic block copolymers in drug delivery: From micellar nanocontainers to biological response modifiers. Crit. Rev. Ther. Drug Carrier Syst..

[38] Mamidi H.K., Rohera B.D. (2021). Application of Thermodynamic Phase Diagrams and Gibbs Free Energy of Mixing for Screening of Polymers for Their Use in Amorphous Solid Dispersion Formulation of a Non-Glass-Forming Drug. J. Pharm. Sci..

[39] Rangel-Yagui C.O., Pessoa A., Tavares L.C. (2005). Micellar solubilization of drugs. J. Pharm. Pharm. Sci..

[40] Steinby K., Silveston R., Kronberg B. (1993). The Effect of Temperature on the Adsorption of a Nonionic Surfactant on a PMMA Latex. J. Colloid Interf. Sci..

[41] Fan W., Zhu W., Zhang X., Xu Y., Di L. (2019). Application of the combination of ball-milling and hot-melt extrusion in the development of an amorphous solid dispersion of a poorly water-soluble drug with high melting point. RSC Adv..

[42] Li S., Tian Y., Jones D.S., Andrews G.P. (2016). Optimising Drug Solubilisation in Amorphous Polymer Dispersions: Rational Selection of Hot-melt Extrusion Processing Parameters. AAPS PharmSciTech.

[43] Censi R., Gigliobianco M.R., Casadidio C., Di Martino P. (2018). Hot Melt Extrusion: Highlighting Physicochemical Factors to Be Investigated While Designing and Optimizing a Hot Melt Extrusion Process. Pharmaceutics.

[44] de Assis J.M.C., Barbosa E.J., Bezzon V.D.N., Lourenço F.R., Carvalho F.M.S., Matos J.R., Araci Bou-Chacra N., Benmore C.J., Byrn S.R., Costa F.N. (2022). Hot-melt extrudability of amorphous solid dispersions of flubendazole-copovidone: An exploratory study of the effect of drug loading and the balance of adjuvants on extrudability and dissolution. Int. J. Pharm..

[45] Nashwan Y.K., Alaa A.A., Moafaq M.G., Saad A.H. (2011). Solubility and dissolution improvement of ketoprofen by solid dispersion in polymer and surfactant using solvent evaporation method. Int. J. Pharm. Sci..

[46] Pantazos I., Poimenidou M., Kouskouridas D., Tzaferas E., Karava V., Cholevas C., Kapourani A., Barmpalexis P. (2025). Influence of Drug Properties, Formulation Composition, and Processing Parameters on the Stability and Dissolution Performance of Amorphous Solid Dispersions-Based Tablets. Polymers.

[47] Yu D., Hoag S.W. (2024). The impact of diluents on the compaction, dissolution, and physical stability of amorphous solid dispersion tablets. Int. J. Pharm..

[48] Ariyasu A., Hattori Y., Otsuka M. (2016). Delay effect of magnesium stearate on tablet dissolution in acidic medium. Int. J. Pharm..

